# Graphene oxide incorporated waste wool/PAN hybrid fibres

**DOI:** 10.1038/s41598-021-91561-0

**Published:** 2021-06-08

**Authors:** Md Abdullah Al Faruque, Rechana Remadevi, Albert Guirguis, Alper Kiziltas, Deborah Mielewski, Maryam Naebe

**Affiliations:** 1 Institute for Frontier Materials (IFM), Deakin University, Geelong, VIC 3216 Australia; 2 Research and Innovation Centre, Ford Motor Company, Dearborn, MI 48121 USA

**Keywords:** Materials science, Chemistry

## Abstract

This work aims to evaluate the potential of using textile waste in smart textile applications in the form of a hybrid fibre with electrical properties. The bio-based electrically conductive fibres were fabricated from waste wool and polyacrylonitrile (PAN) via wet spinning with different wool content. The control PAN and hybrid fibre produced with the highest amount of wool content (25% w/v) were coated with graphene oxide (GO) using the "brushing and drying" technique. The GO nanosheets coated control PAN and wool/PAN hybrid fibres were chemically reduced through hydrazine vapour exposure. The Fourier transform infrared spectroscopy showed the presence of both protein and nitrile peaks in the wool/PAN hybrid fibres, although the amide I and amide A groups had disappeared, due to the dissolution of wool. The morphological and structural analysis revealed effective coating and reduction of the fibres through GO nanosheets and hydrazine, respectively. The hybrid fibre showed higher electrical conductivity (~ 180 S/cm) compared to the control PAN fibres (~ 95 S/cm), confirming an effective bonding between the hydroxyl and carboxylic groups of the GO sheets and the amino groups of wool evidenced by chemical analysis. Hence, the graphene oxide incorporated wool/PAN hybrid fibres may provide a promising solution for eco-friendly smart textile applications.

## Introduction

The growing rate of electronic wastes (e-waste) is accelerating very fast, nearly 3–5% per year, and it is predicted that by the year 2021, this generation of e-waste would be approximately 52.2 million tons^1^. Therefore, the government, industry and academic researchers are focusing on the development of degradable but effective electronic devices using a green and sustainable fabrication process to overcome the environmental issues created by the e-wastes^2^. The selection and utilisation of bio-based agricultural waste materials to produce electronic devices are one of the prime aspects^3^. In production of electronic devices, the development of electrically conductive fibres has led to the fabrication and utilisation of textiles in smart applications (E-Textiles). Compared to the metal-based conductors, the textile-based smart materials are superior due to the unique characteristics such as lightweight, flexibility, and resistance to biological–chemical corrosive materials^4–8^. Several research works have already reported the production of electrically conductive fibres using both natural and petroleum-based resources^4,6,9^. However, due to the environmental concern of disposal and recycling of petroleum-based synthetic fibres e.g., glass and carbon fibres, researchers are becoming more interested in the fabrication and application of nature-based conductive fibres than that of their synthetic counterparts^4,10,11^. Natural fibres are abundantly available, biodegradable, biocompatible, renewable, and possess high surface areas to mass ratio and low density^12–15^. Hence, over the last few years, natural fibre-based conductive fibres have been used in insulating materials, packaging, furniture, automotive, protective, and construction industries^13,16^.

Natural fibres, originated from plant and animal-based sources, are not electrically conductive. Hence, various approaches have been considered to fabricate the natural fibre-based conductive materials, for example, (i) spraying or coating the fibres with different conductive polymers (PEDOT:PSS, polyaniline, polypyrrole), (ii) application of several carbon fillers (carbon fibres, carbon nanotube, carbon black), and (iii) inserting metals and nanowires into the fibrous structure^5,9,16,17^. Another approach to fabricate conductive fibres is immersing the textile fibres, yarns, or fabrics into hydrophilic graphene oxide (GO) solution with oxygen-related functional groups across the graphitic basal planes or in conductive inks^9,18^. Until now, a few studies reported the fabrication of conductive fibres using natural fibres such as cotton, silk, flax, or regenerated cellulosic fibres^9,19–22^. In one study, cotton fabric was coated with GO by the conventional "dip and dry" method, then chemically reduced to fabricate the electrically conductive cotton fabric for the potential smart and E-textiles application^9^. Xia and Lu demonstrated the formation of nature-based composite conductive fibres by in situ chemical polymerisation between the silk fibroin and conductive polymers, which showed better electrical conductivity and enhanced thermal stability^19^. Javed et al. revealed the process of coating the wool and cotton fabric with GO nanosheets by the conventional "brushing and drying" technique and then reduced by UV irradiation to produce an electrically conductive fabric with excellent UV shielding for anti-electrostatic gloves^20^. In another work, cotton fabric was covered with the GO sheet and then thermally reduced to fabricate the conductive cotton fabric to be utilised in strain sensor application^21^. However, a little attention has been drawn to the investigation concerning the potential fabrication of the electrically conductive wool-based fibres using GO materials^12,23^. As GO materials are hydrophilic because of their epoxy, carboxyl, hydroxyl, and carbonyl functional groups, it is expected that these groups can easily create strong hydrogen bonds and covalent bonds with the amino and hydroxyl groups of the wool fibres^20,24,25^, which could be a benefit for the fabrication of the wool-based electrically conductive fibres for various applications.

Hence, this research work aimed to recycle and reuse the waste wool fibres (which are short and non-spinnable fibres and remain unused or are throwing away after the textiles production) with two main objectives. First, fabricating the bio-based hybrid fibres with improved mechanical properties using the maximum proportion of waste wool. Second, the fabrication and characterization of the GO incorporated wool-based conductive fibres. Although GO coating on wool fabric has been used to produce electrically conductive fabric^20^, to the best of our knowledge, there is no report on using waste wool fibre to fabricate the electrically conductive hybrid fibres. The rheology of the dope solutions, morphology, chemical structure, crystallinity, and mechanical properties of the fibres were investigated. The coating of the wool/PAN hybrid fibre with GO and the subsequent chemical reduction was further characterized by SEM, FTIR, Raman, XRD, mechanical, and electrical properties.

## Experimental details and characterisation techniques

### Materials

The textile waste wool fibres were received from the Commonwealth Scientific and Industrial Research Organisation (CSIRO), Geelong Waurn Ponds, Australia. Polyacrylonitrile (molecular weight of 150,000 g/mol), expandable graphite, concentrated sulfuric acid (H_2_SO_4_, 98%), potassium permanganate (KMnO_4_), hydrogen peroxide (H_2_O_2_, 30%), hydrochloric acid (HCl, 32%), sodium hydroxide pellets (NaOH) and hydrazine monohydrate (N_2_H_4_.H_2_O) were purchased from the Sigma-Aldrich, Australia. Dimethyl sulfoxide (DMSO) was procured from Ajax Finechem, Thermo Fisher Scientific. All the chemicals were in analytical grade and used as received without any further purification process.

### Dissolution of wool fibre in an alkaline organic solvent

To dissolve the waste wool fibres into the alkaline organic solvent, the fibres were converted into spray-dried powder. Then the wool spray-dried powder was dissolved into the alkaline NaOH/DMSO solution (as described in the Supplementary Materials Section I).

### Preparation of dope solution and wet spinning

The control PAN and wool/PAN dope solutions (with different wool content of 5%, 15% and 25% (w/v)) were prepared by keeping the proportion of PAN/DMSO solution constant [18% (w/v)]. The wet spinning of the control PAN and wool/PAN (WP) hybrid fibres were carried out using the Dissol (Dissol Pty. Ltd.) wet spinning line. As the blending ratio of wool and PAN was 5:95, 15:85, and 25:75, the corresponding WP hybrid fibres were named WP (5:95), WP (15:85), and WP (25:75), respectively. The detail experimental procedures of these steps are discussed in the Supplementary Materials Section II.

### Conductive hybrid fibres based on graphene oxide (GO) solution

The GO solution was synthesised according to the modified Hummers method, discussed in the Supplementary Materials Section III. The control PAN and WP hybrid fibres produced with 25% (w/v) wool, was coated with the GO solution using the "brushing and drying" method (asserted in the Supplementary Materials Section IV). The electrical conductivity of the GO-coated fibres was boosted through the hydrazine monohydrate vapour exposure that is discussed in the Supplementary Materials Section V.

### Characterisation techniques

The viscosity of all the dope solutions was determined using a rheometer (The Discovery HR-3, TA Instruments, USA). The diameter of the geometry was 40 mm with a 2 cone angle and the truncation gap was 49 µm^26^. The measurement was performed at 25 °C and the shear rate was between 0.1 and 100 s^−1^. The Power-law equation (Eq. S1) was used to calculate the flow behaviour of dope solutions. The longitudinal and cross-sectional morphology of all the fibre samples was observed with a Jeol NeoScope (JCM-5000) and Zeiss Supra 55VP scanning electron microscope (SEM), respectively keeping the accelerating voltage constant at 10 kV. In both cases, the Leica EM ACE600 gold coater was used to coat the fibres before imaging. The Fourier-transform infrared (FTIR) spectra analysis of all the fibres was completed under the Attenuated Total Reflectance (ATR) mode using the Vertex 70 (Bruker, Germany) spectrometer with a scan resolution of 4 cm^−1^ and 32 scans per sample between 400 and 4000 cm^−1^. Data was collected after baseline correction by OPUS 5.5 software. The Raman spectra of the fibres were obtained using a Renishaw InVia Raman microscope (Gloucestershire, UK), equipped with a 514 nm laser and a thermoelectrically cooled CCD detector. The laser power was 5 mW with 3 accumulations and 30 s exposure time. A 50×-objective microscope lens was used to focus on the surface of the fibre sample. The crystallinity of all the fibre samples was analysed at room temperature by X-ray diffraction (XRD) technique (X'Pert Powder, PANalytical, Netherlands) where the operating voltage and the current flows were 40 kV and 30 mA, respectively. The measurement was taken between 6° and 40°; step size was 0.013° and 250 s per step. The number of graphene layers of the as-prepared GO was estimated through XRD analysis by correlating the crystallite size (L) with the interlayer spacing (d spacing) between the graphitic basal plans using Equation S2, S3, and S4 as explained in the Supplementary Materials^27^. The crystallinity index (Cr.I.) of the fibres was calculated by Eq. S5^10^. The single-fibre tester, Favimat + AIRobot2, Textechno, Germany, was used to test the mechanical properties of all of the fibres based on the ASTM C-1557 14^28^. A load cell of 210 cN, a gauge length of 20 mm, and a test speed of 8 mm/min were accepted for performing the tests. The instrument calculated the linear density (tex) of the fibres automatically by applying a vibration resonance technique. Before conducting the tensile test, the samples were conditioned for 48 h in a standard condition of 20 ± 2 °C and 62 ± 2% relative humidity. Twenty-five single fibres were tested for each set of fibres and the average was reported with the standard deviation. The electrical resistance of the control PAN/GO (CPGO), control PAN/reduced GO (CPrGO), wool/PAN/GO (WPGO) and wool/PAN/reduced GO (WPrGO) hybrid fibres was measured via a four-point probe method using a digital multimeter (Keysight, model 34461A). The resistivity of the fibre samples was measured 20 times and the electrical conductivity of the fibres was calculated utilising the formula shown in Eq. S6^19^.

## Results and discussion

### Characterization of the wool/PAN hybrid fibres

#### Rheological properties

**Figure 1:**
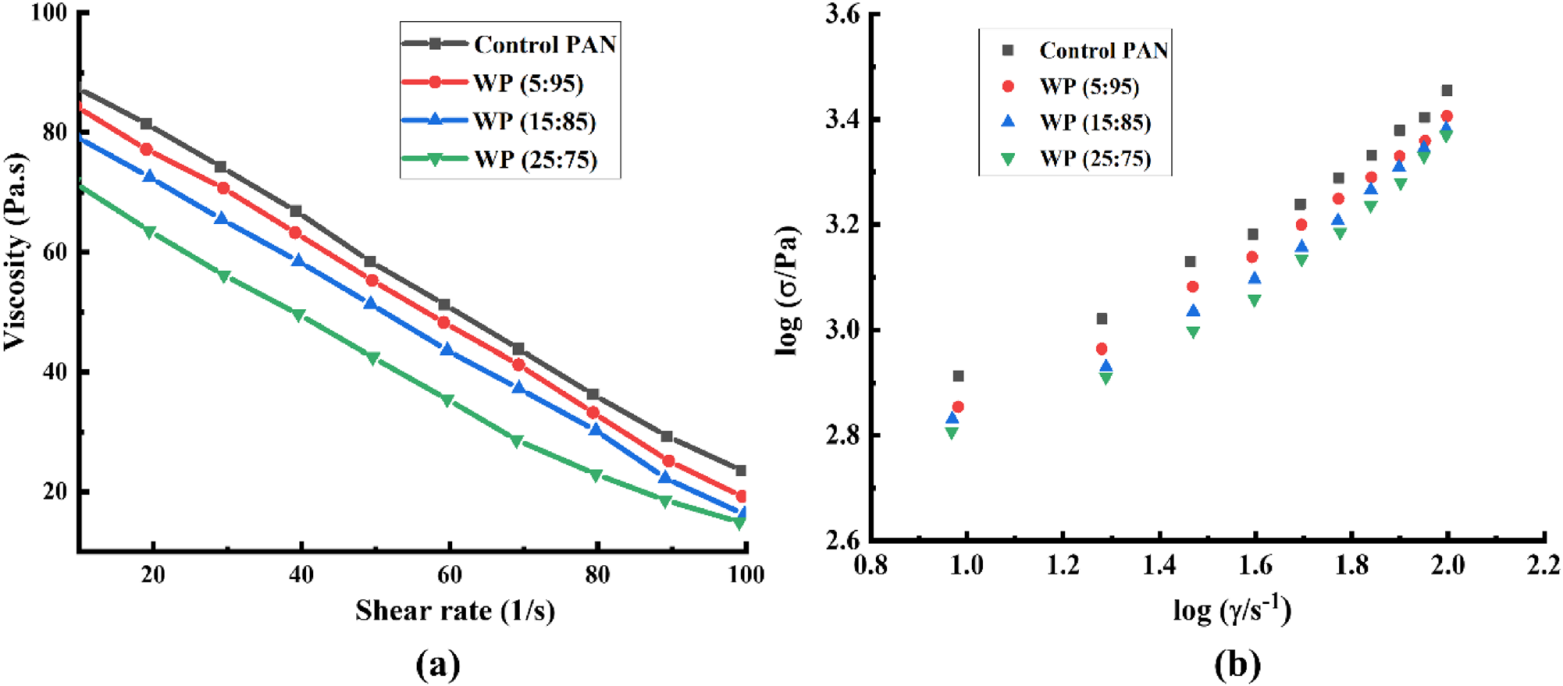
Viscosity vs shear rate **(a)** and logarithmic plots of shear stress vs shear rate **(b)** of the control PAN and wool/PAN blended dope solutions.

The rheology of control PAN and wool/PAN blended dope solution is shown in Fig. 1a. The control PAN dope solution exhibited the highest viscosity rather than all the wool/PAN blended dope solution, with a gradual deterioration in viscosity with the increment of wool content in the dope solution. The increase or decrease in viscosity while adding fillers into the PAN/DMSO dope solution has been reported previously^10,26,29^. It has been found that the addition of fillers such as carbon black, carbon nanotubes, or poly (methyl methacrylate) (PMMA) to the PAN/DMSO increased the viscosity whereas the incorporation of alpaca, lignin, and soy protein into the PAN/DMSO resulted in the viscosity reduction^10,26,29–31^. Fillers such as carbon black and carbon nanotubes assisted in the enhanced molecular orientation of the internal chains. Therefore, a strong internal interaction between the polymeric network prevented the free-flowing movement of the internal chain and increased the dope solution viscosity^29–31^. In contrast, it seems that other fillers such as alpaca, lignin, and soy protein weakened the molecular network and reduced the viscosity of the dope solution^10,26,28^. Similarly, in this work, as the wool was completely dissolved in the alkaline DMSO solution, the protein chain of wool disrupted (as shown by FTIR). Thereafter, while the wool solution was mixed with the PAN/DMSO solution, the polymeric chain of PAN failed to regenerate a well-defined molecular network and eventually reduced the viscosity of the wool/PAN blended dope solutions. Additionally, Fig. 1a shows that with the increased shear rate, the viscosity of the dope solutions falls revealing the existence of the shear-thinning behaviour^26^. In the case of the lower shear rate, the breaking of larger molecular entanglements in a solution is confronted by the growth of newer molecular tangles^32^. Nevertheless, at a greater shear rate, the stability of this molecular restoration is delayed and the boundaries between the molecular falling-off caused lower viscosity of the polymeric solution, which ensures the shear-thinning behaviour of the solution^32^. Although all the dope solutions exhibited shear thinning behaviour, it would not adversely affect the wet spinning process of the fibres, as observed before in the case of lignin/PAN and alpaca/PAN dope solutions^26,28^. The power-law relationship was utilised to further investigate the shear-thinning tendency and the spinnability of the dope solutions. The logarithmic curve of shear stress and shear rate is illustrated in Fig. 1b. Moreover, the consistency index (K), non-Newtonian index (n), and correlation coefficient (R^2^) are calculated and tabulated in Table S1. As per the power-law of fluids, it is well-known that whenever the value of the non-Newtonian index (n) of a solution is greater than 1, the solution represents the shear thickening behaviour, and the solution would be considered as a shear-thinning if the value of the non-Newtonian index (n) is lower than 1^28^. As from Table S1 it can be seen that the value of the non-Newtonian index (n) for all of the dope solutions ranges between 0.58 and 0.51, it can be claimed that all the dope solutions are non-Newtonian fluid, and showing shear thinning behaviour, which is supported by the previous findings of our group^10^. On the other hand, control PAN dope solution exhibited higher value of non-Newtonian index (n = 0.58) compared to the other wool/PAN blended dope solutions, and with the addition of higher amount of wool into the dope solutions the value of the non-Newtonian index reduced from 0.56 to 0.51. As the higher value of n represents the higher spinnability of the dope solutions, it can be claimed that the control PAN dope solution possessed higher spinnability than that of the wool/PAN blended dope solutions^28^.

### Surface morphology

**Figure 2:**
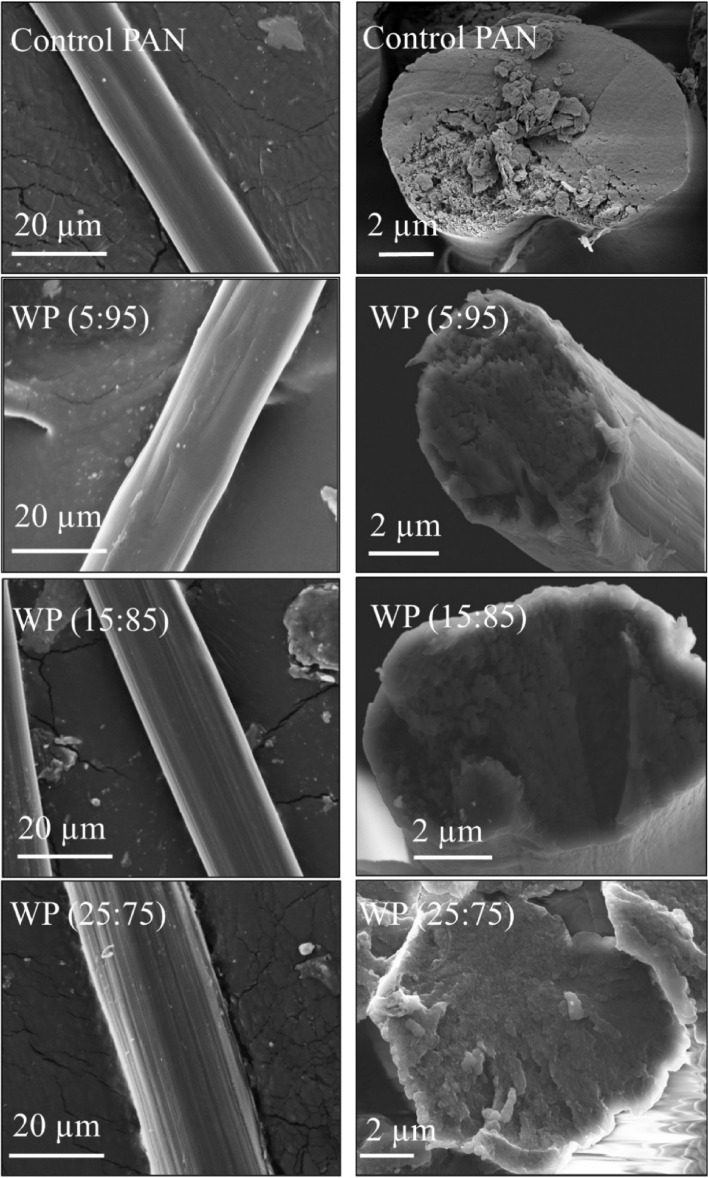
Longitudinal and cross-sectional images of the control PAN and wool/PAN (WP) wet-spun fibres.

The surface and cross-sectional morphologies of the control PAN and WP hybrid fibres are presented in Fig. 2. During the wet spinning of PAN fibres, when the spinning solution comes to the precipitation bath, it solidifies due to the counter diffusion mechanism, which is an outward movement of the solvent from the spinning solution into the coagulation bath and the inward movement of the non-solvent (water) into the polymeric solution from the coagulation bath^10,28^. However, if only water (without any solvent) is used in the coagulation bath, it generates voids and porous areas onto the fibre surface and cross-section, which eventually deteriorate the fibres mechanical properties^10^. Hence, a mixed coagulation bath of solvent (DMSO) and non-solvent (water) was used while wet spinning the PAN fibres. The control PAN fibre indicated its characteristic ribbon-like structure, smooth fibre surface with some traces of striations and typical bean-like cross-section, which might be the result of the counter-diffusion mechanism (Fig. 2)^10,26,28^. The originated surface might be due to the presence of DMSO in the coagulation bath, which results in the sulfonation as reported previously by Kruchinin et al.^33^. In the case of the WP hybrid fibres, a similar trend of smoother fibre surface with the presence of striations was evident, due to the analogous coagulation bath (DMSO: water). All the hybrid fibres produced with wool content showed almost circular cross-section without any noticeable voids and porous areas. During mixing stage and preparing the wool/PAN blended dope solution, the dissolved wool and PAN solution possessed good miscibility. In addition, the observed cross section with no noticeable voids and porous areas further supports the proper dissolution of the wool powder into the NaOH/DMSO solvent, otherwise the micron and nano-sized pores might have been observed in the fibres cross-section as reported earlier^10^. With the addition of a higher amount of fillers into the PAN solution, both the fibre diameter and traces of the striations increases^10,28^. In the cases that addition of filler reduces the viscosity of the spinning solution, and when the solution encounters the coagulating agent, the filler swells quicker than that of the pure PAN spinning solution, and very often results in formation of voids and porous areas into the fibre cross-section^10,26,28^. Moreover, due to the existence of the filler, the fibres cannot be stretched and drawn with the same ratio as the pure PAN solution, neither into the coagulation bath nor into the washing and drying baths. The limitation in applying high draw ratio to the hybrid fibre will negatively affect the fibres and result in mechanical properties reduction compared with the pure PAN fibres.

### Chemical structure and crystallinity analysis

**Figure 3:**
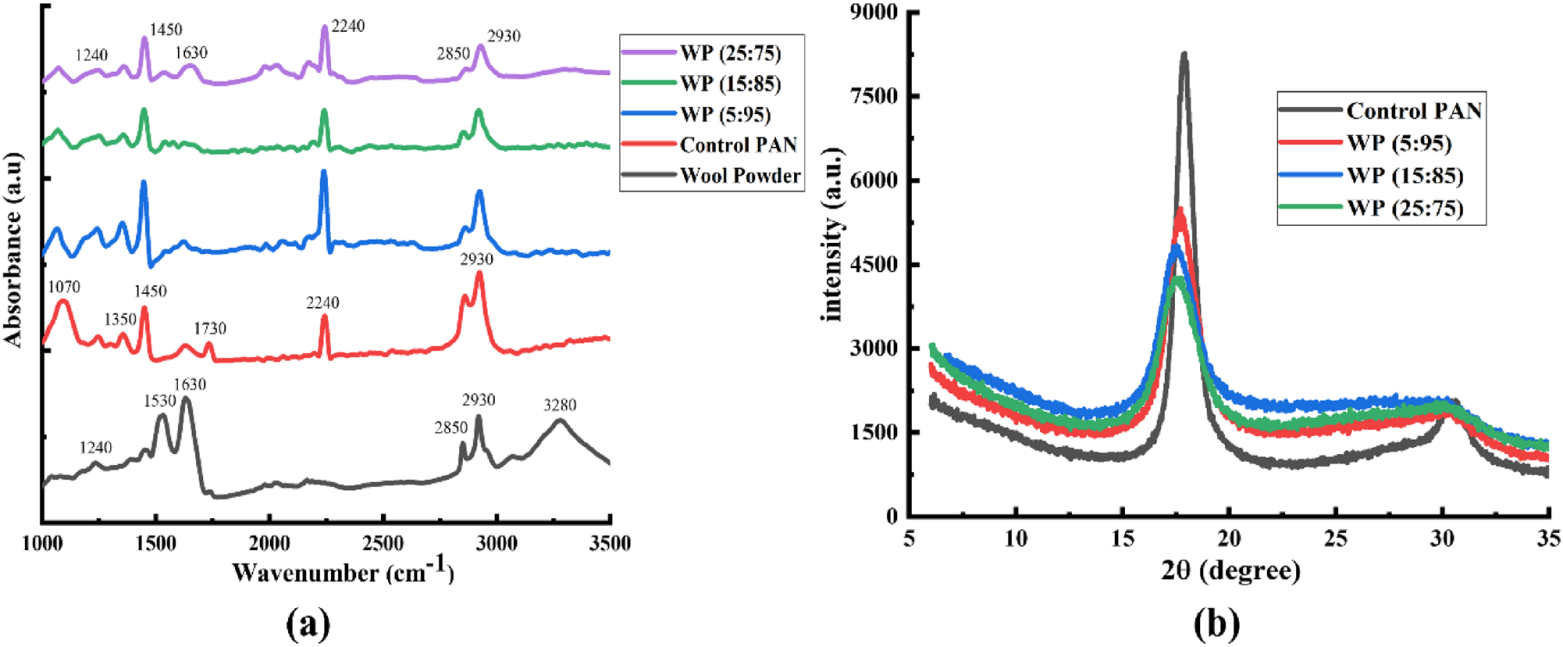
**(a)** FTIR analysis of wool powder, control PAN and wool/PAN (WP) hybrid fibres and **(b)** diffraction pattern analysis of the control PAN and wool/PAN (WP) hybrid fibres.

The Fourier-transform infrared spectroscopy (FTIR) was used to investigate the chemical structure of the wool powder, and wet-spun fibres as shown in Fig. 3a. The wool powder exhibited its characteristic peaks at 1240 cm^−1^, 1530 cm^−1^, 1630 cm^−1^, and 3280 cm^−1^ which specify the presence of the amide III, amide II, amide I, and amide A (N–H stretching and O–H stretching) areas of the protein structure, respectively^10,12^. The robust peaks positioned at 1530 cm^−1^ and 1630 cm^−1^ are due to the methyl C–H deformation and C=O stretching, which indicates the amide II and amide I, respectively. Similarly, the peaks at 2850 cm^−1^ and 2930 cm^−1^ demonstrate the occurrence of symmetric and asymmetric C-H stretching vibrations of the methylene group, respectively^34^. It can be found that although the wool fibre was mechanically converted into powder, neither deterioration in functional groups nor the generation of new peaks occurred, confirming the sustainable conversion into powder from fibres. The control PAN fibre represented its primary functional peak at 2240 cm^−1^ attributed to the C≡N (nitrile group) and the secondary peaks at 1470 cm^−1^ and 2930 cm^−1^ are credited to the bending and stretching vibration of methylene groups, correspondingly^26^. The other functional groups have been found at 1070 cm^−1^, 1350 cm^−1^, and 1730 cm^−1^ because of the presence of C–CN groups, stretching of C–H in CH groups, and specific stretching and absorption of C=O in COOH groups, respectively^35–38^. For the WP hybrid fibres prepared with different weight ratios of wool content, it can be seen that amide-II (1530 cm^−1^), amide-A (3280 cm^−1^) groups from the wool powder have been diminished, which might be due to the proper dissolution of the wool powder in the alkaline organic solution^39^. These findings suggest the proper dissolution of wool as well as accurate blending and mixing of the wool solution and PAN solution while preparing the wool/PAN blended dope solution. On the other hand, the diffraction patterns of the control PAN and WP hybrid fibres are shown in Fig. 3b and the crystallinity index (Cr.I.) that was calculated by Eq. S5 is tabulated in Table S2. The diffraction pattern of the control PAN fibre exposed a typical primary high-pitched 2θ peak at 17.5 and the other secondary small-intensity 2θ peak at 30.5^40^. The presence of the crystalline region in the fibrous material results in a sharper peak and the amorphous region of the fibre causes the halo or smaller peaks^10,26,28^. From Fig. 3b, it can be seen that the control PAN fibres exposed the sharpest primary 2θ peak at 17.5, which is higher than that of the other WP hybrid fibres that indicating the presence of the higher crystalline and lower amorphous regions in the control PAN fibres rather than the WP hybrid fibres. This might be due to the addition of wool into PAN, as it has already been established that with the incorporation of fillers, the crystallinity of the PAN fibres reduces while the amorphousness increases^10,26^. A similar phenomenon was found while calculating the crystallinity index of all the fibres (Table S2). The control PAN fibres showed a crystallinity index of 75% while this was gradually reduced to nearly 67%, 57%, and 52% in the case of the WP hybrid fibres prepared with 5%, 15%, and 25% (w/v) wool content, respectively. The results found in this study support the outcomes of the related studies, where different filler materials were used with PAN to fabricate the wet-spun PAN-based hybrid fibres^10,26^.

### Mechanical properties

The stress vs. strain curve, and the average values of strength (MPa), strain (%), diameter (µm), and linear density (tex) of the control PAN and the WP hybrid fibres are presented in Fig. S4 and Table 1, respectively. The control PAN fibres exhibited a lower diameter (~ 16 µm) and higher strength (~ 438 MPa) compared to all other WP hybrid fibres. With the incorporation of wool into the PAN system, the fibre diameter of the WP hybrid fibres produced with 5%, 15%, and 25% (w/v) wool content gradually increased (ranging from ~ 20 to ~ 33 µm) and the strength dramatically declined (between ~ 342 and ~ 116 MPa). The addition of fillers to the PAN increases fibre diameter and amorphous regions. While the increased crystallinity eventually ensures the unidirectional molecular orientation within the fibres, this alignment is hampered upon the reduction of crystallinity and enhancement in amorphousness of the fibres^28,41^. Therefore, changes in mechanical properties are expected. Fibres, especially the hybrid fibres, with a relatively larger fibre diameter show lower strength, as these fibres possess higher inner surface defects compared to the finner fibres^41^. Hence, with the higher wool content, the fibre diameter increased and the number of defects of the fibres inner surface increased, and ultimately the fibre broke and showed lower mechanical properties compared to the fibres with lower wool content and control PAN. However, the strength of the wet-spun fibres can be enhanced by introducing "drawing and stretching" to the fibres. As one of the main focus of this research work was to fabricate bio-based hybrid fibres by incorporating the higher amount of natural waste materials, the WP hybrid fibre fabricated with 25% wool was stretched to enhance the mechanical properties of this fibre following the process described before^28^. It can be found that the fibre strength improved more than 60% (from ~ 116 to ~ 327 MPa), and the fibre diameter reduced up to ~ 70% (from ~ 30 to ~ 20 µm), which might be due to the applied drawing and stretching (Table 1). By applying this modification on the fibres, the defects of inner fibre surface reduced and the molecular orientation of the polymeric chain of the fibre changed, which ultimately enhanced the fibre mechanical properties of the fibres produced with 25% wool content (WP 25:75). The modified fibre exhibited higher mechanical strength (~ 327 MPa) compared to the other fibres such as feather barbs (~ 161 MPa), keratin fibres (~ 138 MPa), wool fibres (~ 173 MPa), alpaca/PAN composite fibres (~ 297 MPa), and viscose fibres (~ 276 MPa), reported in the literature^28,42^. Therefore, this fibre (WP 25:75) with enhanced mechanical properties was further coated with the GO to fabricate the bio-based electrically conductive fibre, which will be discussed in the next section.

**Table 1:** Mechanical properties of all the wet-spun fibres with the standard deviation.

Sample	Diameter (µm)	Linear density (tex)	Strength (MPa)	Strain (%)
Control PAN	16.96 ± 1.74	0.16 ± 0.01	438.32 ± 1.96	8.66 ± 0.76
WP (5:95)	19.84 ± 1.41	0.18 ± 0.02	342.83 ± 2.53	11.37 ± 0.68
WP (15:85)	23.42 ± 2.05	0.24 ± 0.02	192.12 ± 1.78	11.62 ± 1.41
WP (25:75)	30.39 ± 2.40	0.33 ± 0.02	116.67 ± 1.95	12.07 ± 1.75
WP (25:75)^a^	20.58 ± 1.60	0.19 ± 0.02	327.28 ± 1.98	10.92 ± 1.39

^a^WP (25:75) hybrid fibre after stretching at 90 °C and drying at 120 °C.

## Characterization of the wool/PAN/GO and wool/PAN/reduced GO hybrid fibres

### Morphology

**Figure 4:**
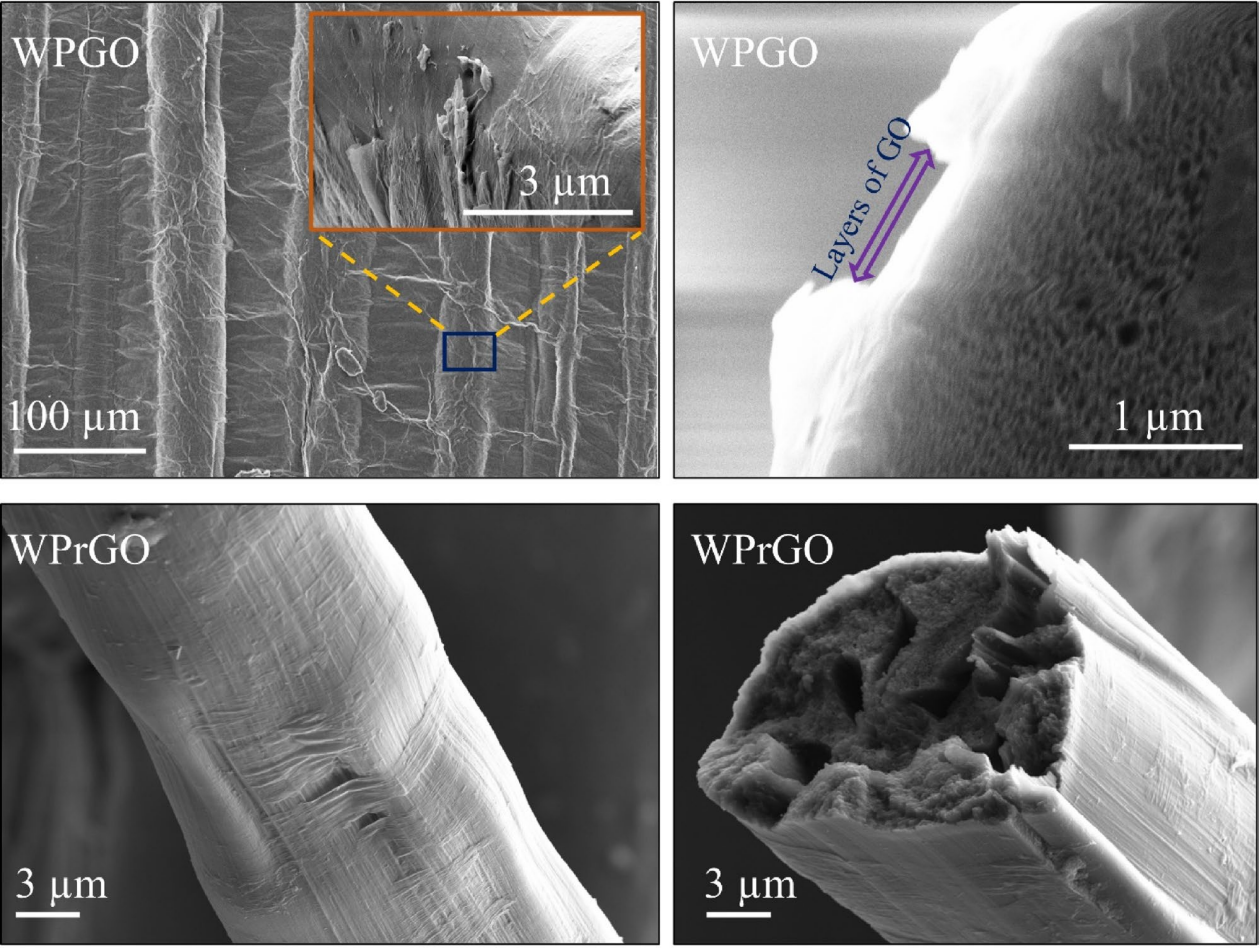
Longitudinal and cross-sectional images of the wool/PAN/GO (WPGO) and wool/PAN/reduced GO (WPrGO) hybrid fibres.

**Figure 5:**
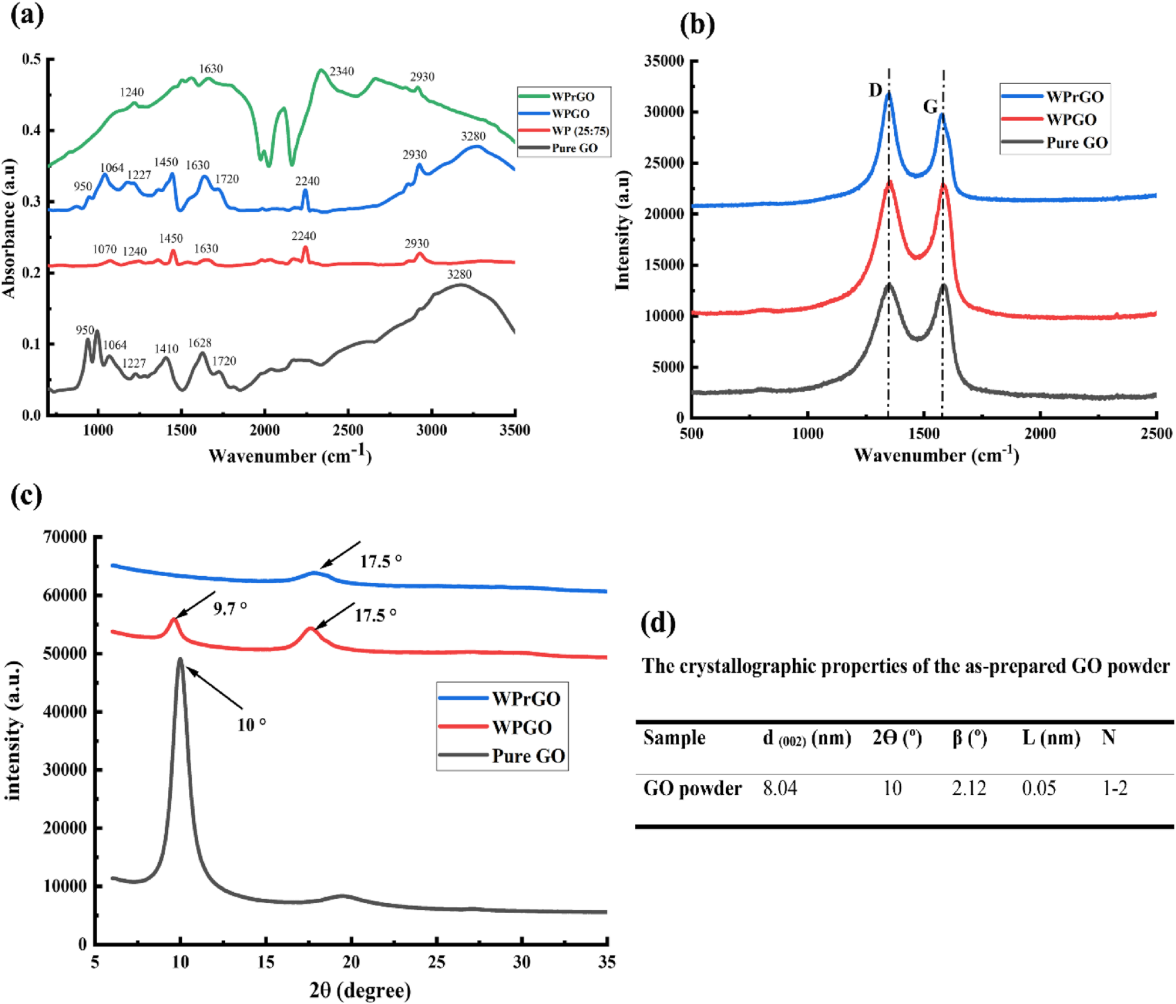
**(a)** FTIR, **(b)** Raman, **(c)** diffraction pattern analysis of pure graphene oxide (GO), wool/PAN/GO (WPGO), and wool/PAN/reduced GO (WPrGO) hybrid fibres, and **(d)** crystallographic properties of the as-prepared GO powder.

The hybrid fibres produced with the highest amount of wool content (WP 25:75) were coated with GO and then chemically reduced using hydrazine monohydrate. The surface and cross-sectional morphology of the WPGO and WPrGO hybrid fibres are presented in Fig. 4. A uniform coating of GO on the fibre surface was observed on the WPGO fibres. It can be seen that the GO was exfoliated into a thin sheet with wrinkles, which might be the result of preparatory flaws of GO from expandable graphite and the presence of oxygen-containing functional groups^43^. Additionally, it can be predicted that GO sheets possibly penetrated the fibre surface uniformly, as both the WP (25:75) hybrid fibre and the GO sheets contained hydroxyl groups, which can further be seen from Fig. 5a (FTIR analysis, next section) where the intensity of hydroxyl group increased in the case of the WPGO fibre compared to the WP (25:75) fibre. Furthermore, as the WP (25:75) fibre is enclosed with ester and carbonyl groups, there is a chance that it can be bonded with GO through the van der Waals forces and hydrogen bonds, respectively, and result in an excellent penetration^20^. On the other hand, the removal of wrinkles like GO thin sheets and spotting of some small clusters of sheets on the fibre surface ensured the effective chemical reduction of the WPrGO fibres.

### Chemical structure and crystallinity analysis

Analysis of the fine chemical structures of the pure GO, WPGO, and WPrGO hybrid fibres was performed using the FTIR, Raman, and diffraction pattern characterisation techniques and showed in Fig. 5 (a–c, respectively). Apart from the basic protein and nitrile FTIR peaks, the WPGO hybrid fibre showed the presence of GO, where the peaks from 950 to 1450 cm^−1^ originated due to the presence of C–O bonds of hydroxyl groups (e.g. phenol), or epoxy groups, and the other C–OH vibrations originated from the COOH groups^44,45^. The other adsorption bands found at 1720 cm^−1^ and from 2800 to 3000 cm^−1^ belonged to the carboxyl (–C=O), asymmetrical, and symmetrical stretching vibration of –CH_2_ groups^20,44,45^. Besides, the presence of hydroxyl groups (–OH) at 3280 cm^−1^ can also be evident as the intensity of this peak position has boosted^44,45^. Hence, it can be predicted that GO was effectively bonded with the –NH_2_, –OH, and C–O functional groups of both wool and PAN through the van der Waals forces and hydrogen bonds (hydrogen atoms from carboxyl groups and oxygen from ester groups)^20^. However, after the reduction process via hydrazine vapour, it can be seen that the oxygen containing functional groups such as epoxy, alkoxy and carboxyl groups diminished leaving behind the typical –CH_2_ groups of GO sheets^44^ along with the protein and nitrile peaks of wool and PAN. These findings suggest the effective reduction of the WPGO to the WPrGO hybrid fibre.

On the other hand, while performing the Raman analysis, the WP (25:75) fibre did not exhibit any Raman peaks (data is not shown), which might be due to the haphazard orientation of the PAN molecules and absence of the carbonaceous materials^46^. However, after coating with GO, the WPGO fibre showed the typical D and G band peaks belong to the GO (Fig. 5b). The D band is generally used to identify the potential defects and edges of the graphene structure whereas the G band efficaciously replicates the actual amount of graphene layers^47,48^. Additionally, the D band originated when the graphene leaves the Brillouin zone due to the vibration associated with the 2D in-plane lattice and the G band is formed owing to the sp^2^ hybridized carbon atoms in a 2D hexagonal lattice^45,47^. Furthermore, the intensity ratio between the D band and G band (I_D_/I_G_) denotes a very close relationship with the nature of defects in graphene, and it determines the efficiency of the reduction of GO nanostructure^45,47^. In this study, the pure GO showed its typical D band and G band peaks at 1349.5 cm^−1^ and 1586.6 cm^−1^, respectively whereas the WPGO hybrid fibre exhibited its D band and G band peaks at 1349.5 cm^−1^ and 1606.1 cm^−1^, respectively. However, after the reduction process, the WPrGO hybrid fibre showed a slight movement of the G band peak at 1576.3 cm^−1^. The intensity ratio (I_D_/I_G_) of the GO, WPGO, and WPrGO hybrid fibres increased from 0.97 to 1.08, respectively. Therefore, as the intensity ratio increased and the oxygen-containing functional groups eliminated, it can be claimed that the GO was successfully reduced, and it will enhance the electrical conductivity of the WPrGO hybrid fibres.

The crystallinity of the as-prepared GO material was further investigated through XRD analysis prior to being utilised in the coating process of the wet-spun fibres. X-ray diffraction patterns of the GO nano-powder, WPGO, and WPrGO fibres were recorded at different 2° angles between 5 and 40 as shown in Fig. 5c. A sharp peak was observed for GO nano-flakes corresponding to (002) plane at 2° equal to 10^27,49^. These planes are corresponding interlayer distance d _(002)_ = 8.0 Å due to the existence of stacked oxygen atoms between graphene sheets during the exfoliation process. The number of graphene layers of the as-prepared GO was estimated by correlating the crystallite size (L) with the interlayer spacing (d spacing) between the stacking graphitic basal plans and the results are shown in Fig. 5d. The GO nano powder showed a number of the graphene layer, nearly 1.1 (a.u.), which indicated that the graphitic powder was successfully exfoliated into a crystalline structure, consisting of few-layer GO sheets^50^. Besides, apart from the primary and secondary 2θ peaks at 17.5 and 30.5, the WPGO hybrid fibre showed an additional 2θ peak at 10, which is the typical XRD peak of the GO (the typical 2θ peak of pure GO ranges between 9.7 and 10.6)^45^. However, upon performing the reduction process, this peak diminished leaving behind the peaks originated from the PAN fibres. Hence, this analysis ensured the effective fabrication of WPGO and WPrGO hybrid fibres.

### Electrical conductivity test

The electrical conductivity test is certainly one of the best approaches to evaluate the efficacy of the reduction of GO. To analyse the effect of the addition of wool to the PAN, both the control PAN and the WP (25:75) fibres were coated with the GO sheets. GO sheets are not electrically conductive due to the lacking of π- conjugated orbital structure^9^. In similar, the CPGO and WPGO fibres showed no conductivity with zero surface resistance (data shown as “Overloaded”). However, after reduction, both the CPrGO and WPrGO fibres showed surface resistivity and electrical conductivity. The surface resistivity of the CPrGO and WPrGO hybrid fibre was found to as 4883.03 ± 1.41 Ω and 1676.10 ± 1.84 Ω and the electrical conductivity was 94.52 ± 0.02 S/cm and 179.35 ± 0.19 S/cm, respectively. This increased electrical conductivity of the WPrGO hybrid fibre than that of the CPrGO fibre further confirmed the efficient bonding of the amino groups of wool with the hydroxyl and carboxylic groups of the GO sheets as shown by chemical analysis (FTIR). Additionally, due to having higher polar nature, the amide groups can easily be bonded with hydrogen or accept hydrogen on both sides of its structure, which helps the enhancement of the electrical conductivity of the WPrGO hybrid fibres^4,9^. Compared to other chemical reduction processes, the use of hydrazine hydrate as a reducing agent increases the electrical conductivity of the material^51^. Therefore, it might also be an effective reason for the increased electrical conductivity shown by the WPrGO fibres. Besides, the electrical conductivity of the WPrGO hybrid fibre was found to be higher than some other reported wet-spun graphene fibres and graphene-coated hybrid fibres, as shown in Table 2. Hence, it can be claimed that the presence of wool increased the potential of electrical conductivity of the graphene-based WP hybrid fibres, due to the effective bonding between wool and GO. This higher electrical conductivity further confirms the effective coating of GO sheets on the WP (25:75) hybrid fibres and the subsequent well-performing chemical reduction of the GO sheets. In addition to the enhanced conductivity, the reduction of GO also increased the mechanical properties of the WPrGO hybrid fibres (as shown in Table 2), which might be owing to the enhancement of degree of order of the graphene sheets onto the fibre surface and presence of effective interlinkage among the graphene sheets originated from the more densely packed stacking of the reduced GO sheets onto the fibre surface^52,53^. Similar results were also reported by the authors who found that the reduction of GO increases the mechanical properties of the fibres due to the closely packed graphene stacks, improved degree of order and better interfacial properties of the fibres onto the fibre surface^53–55^. The fabricated WPrGO hybrid fibres have a great potential in applications such as energy, sensing, and separation^56^. This highly conductive fibre with improved strength can find its potential application in the automobile industry replacing metals with the textile fibres, which are used to impart the electrical conductivity in the heated car seat^57^. Moreover, the WPrGO hybrid fibres can be used as supercapacitors, to replace the graphene/polypyrrole hybrid fibres supercapacitors with the electrical conductivity 141 S/cm^58^.

**Table 2:** Electrical conductivity of selected graphene and graphene-based hybrid fibres reported in literature.

Fibres	Reduction process	Strength (MPa)	Electrical conductivity (S/cm)	Reference
Graphene fibres	Thermal annealing at 2850 °C	940	2210	^59^
Graphene fibres	Thermal annealing at 3000 °C	–	7700	^60^
Graphene fibres	Hydroiodic acid (40%)	182	35	^54^
Graphene/polypyrrole fibres	Hydroiodic acid (40%)	80	1.37–1.44	^61^
Graphene/polypyrrole fibres	Ascorbic acid	–	141	^58^
Graphene/carbon nanotube	Chemical reduction	84–165	102	^62^
Graphene nanoribbon (GNR)/ Kevlar fibres	–	–	20	^63^
Graphene/polyglycerol fibres	Hydrazine monohydrate	42	2.44	^64^
Wool/PAN/reduced GO hybrid fibre (WPrGO)	Hydrazine monohydrate	488	180	This study

## Conclusion

In this study, the alkaline organic solvent was prepared to dissolve the waste wool fibre, which was then blended with PAN to fabricate the wool/PAN dope solution to be wet spun into wool/PAN (WP) hybrid fibres. The hybrid fibres showed a smoother fibre surface without any voids and porous cross-section along with the presence of both protein and nitrile functional groups through the SEM and FTIR analyses, respectively. A gradual deterioration in the crystalline structure of the hybrid fibres with the increment of wool content was evident by the XRD analysis. Similarly, the fibre diameter increased, and strength decreased in the case of the WP (25:75) fibre, which was produced with a higher amount of wool content (25%). However, a significant reduction in the fibre diameter and increase in the strength of the WP (25:75) fibre was achieved by applying drawing and stretching technique. The WP (25:75) hybrid fibre was then coated with GO sheets by a simple "brushing and drying" technique, and chemically reduced using hydrazine monohydrate vapour exposure to enhance the electrical conductivity of the fibres. Through the morphological, chemical structure, and diffraction pattern analysis, it was found that the coating of the WP (25:75) hybrid fibres with GO and the subsequent chemical reduction was performed in an appropriate way, which was further confirmed by the electrical conductivity test. The wool/PAN/reduced GO hybrid fibre (WPrGO) showed an electrical conductivity of ~ 180 S/cm, higher than that of control PAN/reduced GO (CPrGO) fibres (~ 95 S/cm). Moreover, the electrical conductivity and mechanical strength of the WPrGO hybrid fibres were found higher than some of the reported pure graphene and graphene-coated hybrid fibres. In this study, the WPrGO hybrid fibre was fabricated using 25% (w/v) wool content, where the wool fibre was collected from the waste sources. This environmentally friendly approach may limit the scope of throwing the wastes into the landfill. Moreover, due to the presence of 25% wool into the WPrGO hybrid fibre, a greater biodegradation ability (weight loss %) is expected compared to the CPrGO produced from 100% synthetic PAN polymer. Hence, it can be predicted that the fabricated hybrid fibre would be able to retain the potential of use in diversified application areas, while supporting the eco-friendliness by reducing the generation of e-waste, reusing the waste materials to fabricate the e-textiles, and lowering the consumption of the petroleum-based PAN.

## Supplementary Information

Supplementary Information.

## Data Availability

The raw/processed data required to reproduce these findings cannot be shared at this time as the data also forms part of an ongoing study.
