# Supplementary material for: Graphene oxide incorporated waste wool/PAN hybrid fibres
Source: Sci Rep. 2021 Jun 8;11:12068. doi: 10.1038/s41598-021-91561-0 (PMC8187707; doi:10.1038/s41598-021-91561-0)
Supplement: Supplementary file 1 — Supplementary Information. [file 41598_2021_91561_MOESM1_ESM.docx]

**Supplementary Materials for**

**Title**

**Graphene oxide incorporated waste wool/PAN hybrid fibres**

**Authors**

Md Abdullah Al Faruque^1^, Rechana Remadevi^1^, Albert Guirguis^1^, Alper Kiziltas^2^, Deborah Mielewski^2^, Maryam Naebe^1^*

**Affiliations**

*^1^Deakin University, Institute for Frontier Materials (IFM), Geelong, Victoria 3216, Australia*

*^2^Research and Innovation Center, Ford Motor Company, Dearborn, 48121, Michigan, USA*

*** Corresponding author:**

E-mail: [maryam.naebe@deakin.edu.au](mailto:maryam.naebe@deakin.edu.au) (Maryam Naebe)

***Section I: Dissolution of wool fibre in an alkaline organic solvent***

Before dissolving the waste wool fibres in the alkaline organic solvent, it was converted into spray-dried powder ^1^. Briefly, the waste wool fibres were washed and dried overnight in an oven at 60 ⁰C. The dried fibres were chopped into snippets using a rotary cutter mill (Pulverisette 19, Fritsch GmbH, Germany) and the snippets were then milled into a slurry using an Attritor mill (2S, Union Process, USA). Finally, the slurry was converted into spray-dried powder through the laboratory-scale mini spray drier ((B-290, Buchi Labortechnik AG, Switzerland). NaOH (1 g) and DMSO (100 ml) were mixed and continuously stirred at 80 ⁰C for 2 hrs, to prepare the alkaline organic solvent (NaOH/DMSO, pH ~12-13). Then 8 g of wool spray-dried powder was dissolved into the alkaline NaOH/DMSO solution and unceasingly agitated at 80 ⁰C for 6 hrs. Fig. S1. showed the digital images of the addition of wool powder in NaOH/DMSO solution (Fig. S1 a), and 5%, 15%, and 25% wool powder dissolution in NaOH/DMSO solution (Fig. S1 b, c, and d, respectively).

***Section II: Preparation of dope solution and wet spinning***

The control polyacrylonitrile (PAN) dope solution [18% (w/v)] was prepared by pouring 18 g PAN polymer into 100 ml dimethyl sulfoxide (DMSO) and stirring (IKA RW20 digital mechanical stirrer) at 100 rpm and 70 °C for 24 hrs. In the case of the 5%, 15% and 25% (w/v) wool/PAN blended dope solution preparation, 0.48 g, 1.60 g, and 3 g of wool spray-dried powder was dissolved in the alkaline organic solution (NaOH/DMSO) and then mixed with the PAN solution respectively, by keeping the proportion of PAN/DMSO solution constant [18% (w/v)]. After mixing, the solutions were stirred continuously using the IKA RW20 digital mechanical stirrer at 100 rpm and 70 °C for 4 hrs to ensure proper mixing and blending of the wool/PAN blended dope solution. As the blending ratio of wool and PAN was 5:95, 15:85, and 25:75, the corresponding wool/PAN hybrid fibres were named WP (5:95), WP (15:85), and WP (25:75), respectively (The digital images of the samples are presented in Fig. S2). The wet spinning of the control PAN and the wool/PAN (WP) hybrid fibres was carried out using the Dissol (Dissol Pty. Ltd.) wet spinning line fitted with a gear pump and consists of a temperature-controlled coagulation bath, a washing zone, a drying zone, and a winding zone. This continuous wet spinning system spins around one litre (1 L) of the dope solution and 3 kilometres of filament in length. The dope solution was extruded at ambient temperature and through the spinneret (100 μm diameter with 100 holes) into the coagulation bath of DMSO and water (65:35). After precipitation, the fibres were passed through the washing bath (room temperature), stretching bath (80 °C) and were collected on a spool at the winding zone. Later, the hybrid fibre produced with the highest amount of wool (25%) was further stretched in the stretching bath of the wet spinning line at 90 °C and then dried in a laboratory oven at 120 °C. The digital image of all the wet-spun fibres is shown in Fig. S3.

***Section III: Graphene oxide (GO) synthesis***

The graphene oxide (GO) was synthesised according to the modified Hummers method ^2^. Briefly, 1 g of expanded graphite and 200 mL of sulphuric acid (H_2_SO_4_) were mixed in a beaker and stirred continuously overnight. Then, 10 g of potassium permanganate (KMnO_4_) was added very slowly with the mixture of expanded graphite and sulphuric acid until the colour of the mixture turned green. Next, 200 mL of de-ionised (DI) water was gradually added to the mixture while the colour of the mixture changed from green to purple to brown. After that, 30 mL of hydrogen peroxide (H_2_O_2_) solution was added dropwise into the mixture until the colour of the solution turned into light yellow. Subsequently, 500 mL of hydrogen chloride (HCl) solution (9:1 v/v, water to HCl) was added into the mixture and stirred for 30 min before it was centrifuged at 6000 rpm for a 20-min duration. Finally, the solution was repeatedly washed around 6-7 times with DI water and centrifuged at 10000 rpm for 20-60 min until the pH reached 7.

***Section IV: Preparation of the Graphene oxide (GO) coated fibres***

The control PAN and wool/PAN (WP) hybrid fibre produced with 25% (w/v) wool, was coated with the graphene oxide solution using the "brushing and drying" technique ^3,4^. Briefly, a specified amount of fibre was placed onto a tray, and the two ends were fixed with adhesive tape. Then, using the brush-coating technique, the fibre was coated with a 1% (w/v) GO solution and dried in air at room temperature for 8 hrs. This step was repeated 3 times to obtain GO coated control PAN and wool/PAN hybrid fibres, which were named as control PAN/GO (CPGO) and wool/PAN/GO (WPGO), respectively.

***Section V: Reduction of the Graphene oxide (GO) coated fibres***

The CPGO and WPGO fibres were reduced using hydrazine monohydrate vapour exposure ^5^. In brief, around 5 cm of fibre was kept on a microscopic slide and inserted into a round bottom flask. 3-5 ml of hydrazine monohydrate was poured into the round bottom flask and heated at 80 °C for 24 hrs to accomplish the chemical reduction of the fibres using hydrazine vapour inside the chamber. After reduction, the fibres were termed as control PAN/reduced GO (CPrGO) and wool/PAN/reduced GO (WPrGO) hybrid fibre.


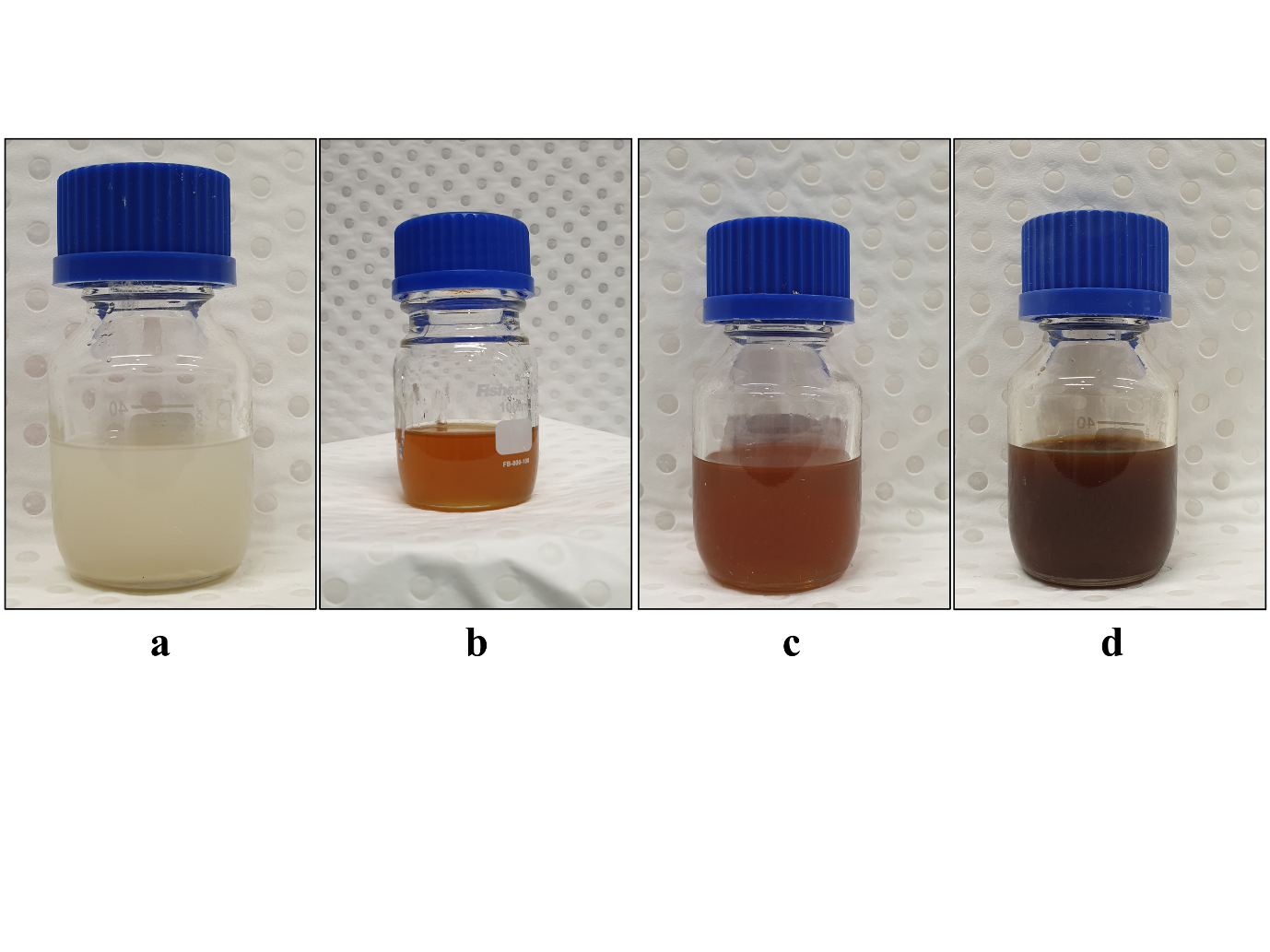


**Fig. S1.** Digital images: (a) Wool powder in NaOH/DMSO solution, (b) 5% wool powder dissolved in NaOH/DMSO solution, (c) 15% wool powder dissolved in NaOH/DMSO solution, and (d) 25% wool powder dissolved in NaOH/DMSO solution.


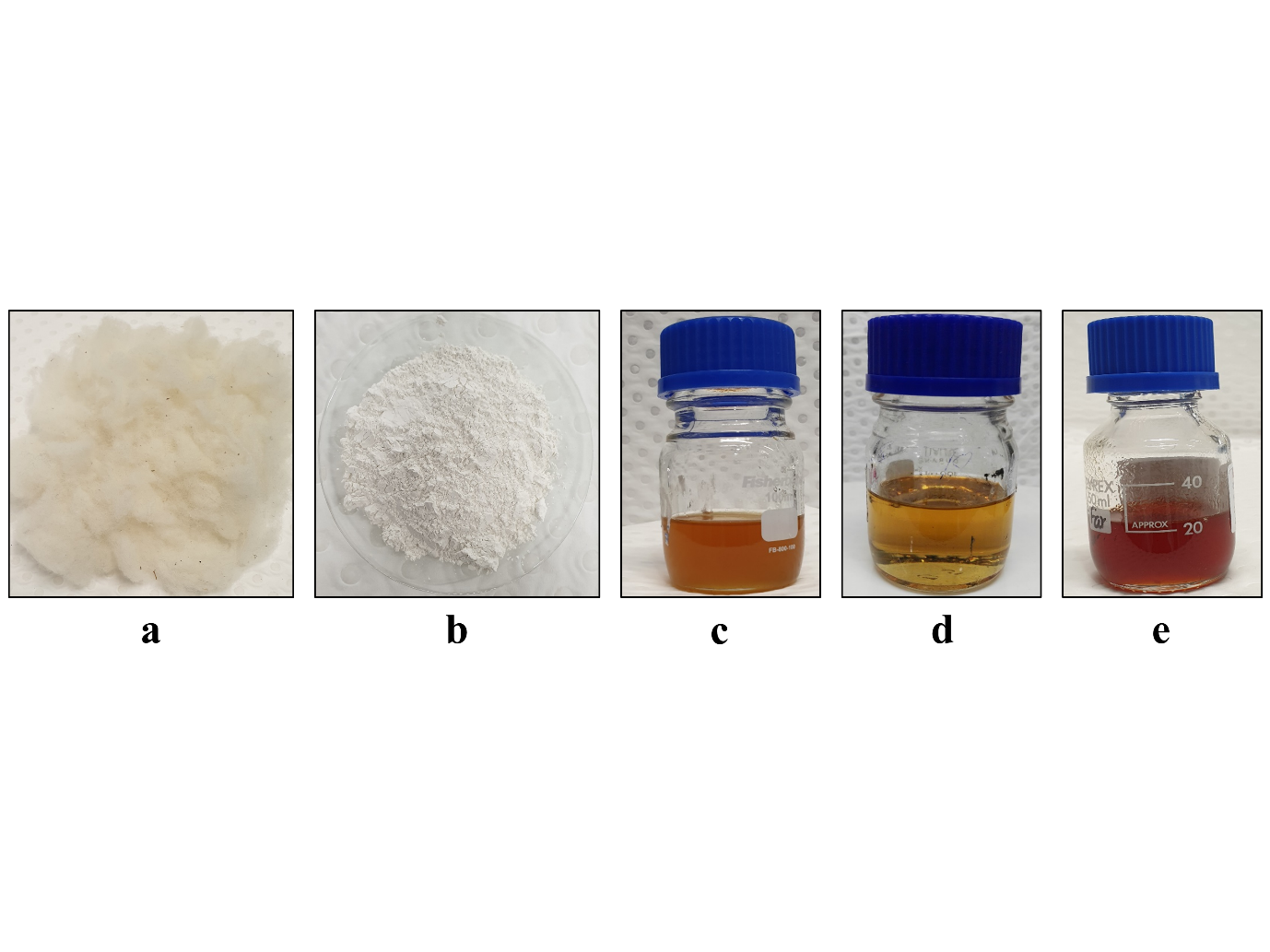


**Fig. S2.** Digital images: (a) waste wool fibres, (b) wool spray-dried powder, (c) dissolved wool (5%) in NaOH/DMSO solution, (d) control PAN and (e) wool/PAN (5:95) blended dope solution.


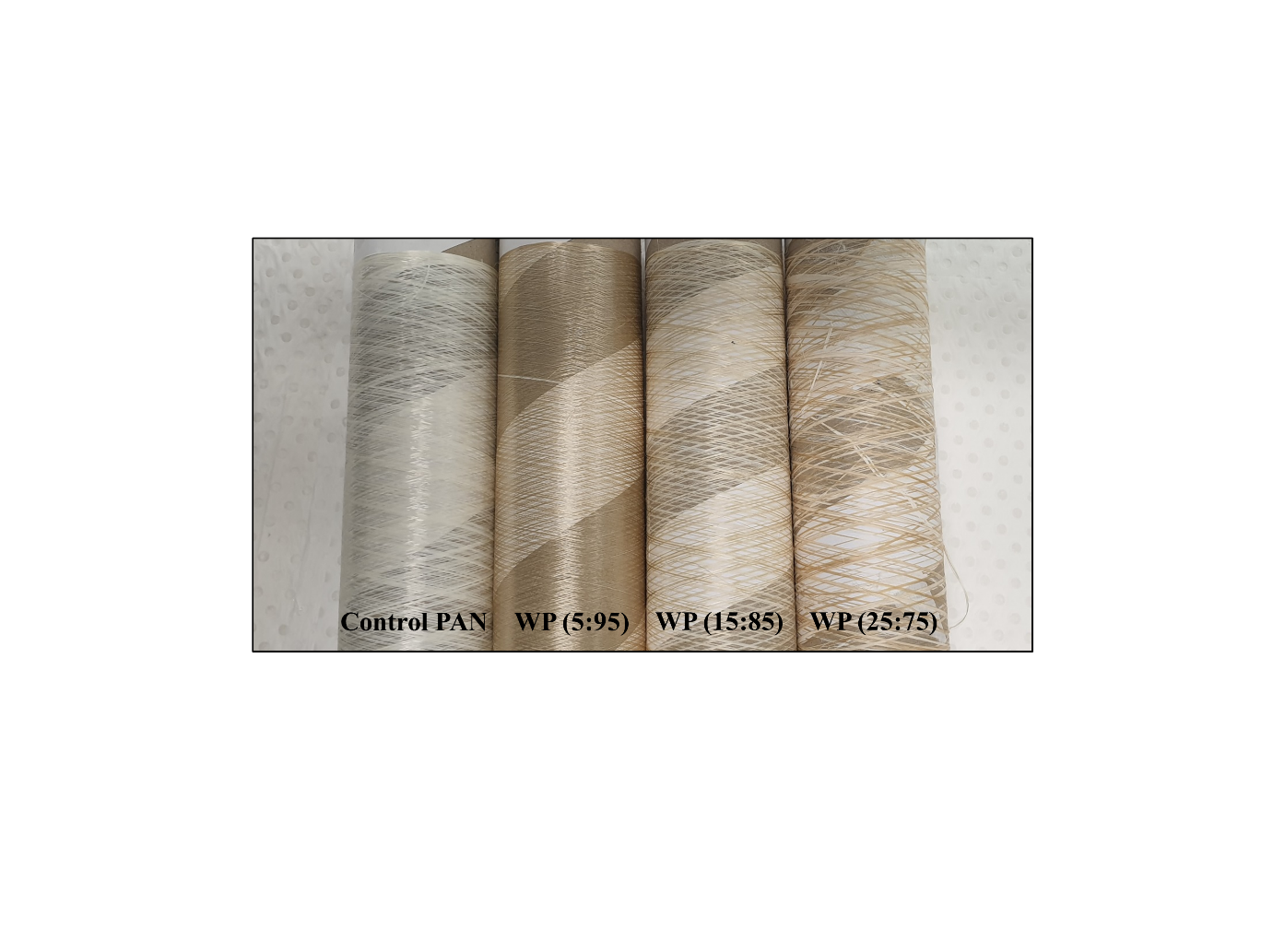


**Fig. S3.** Digital image of the control PAN and the wool/PAN (WP) hybrid fibres.


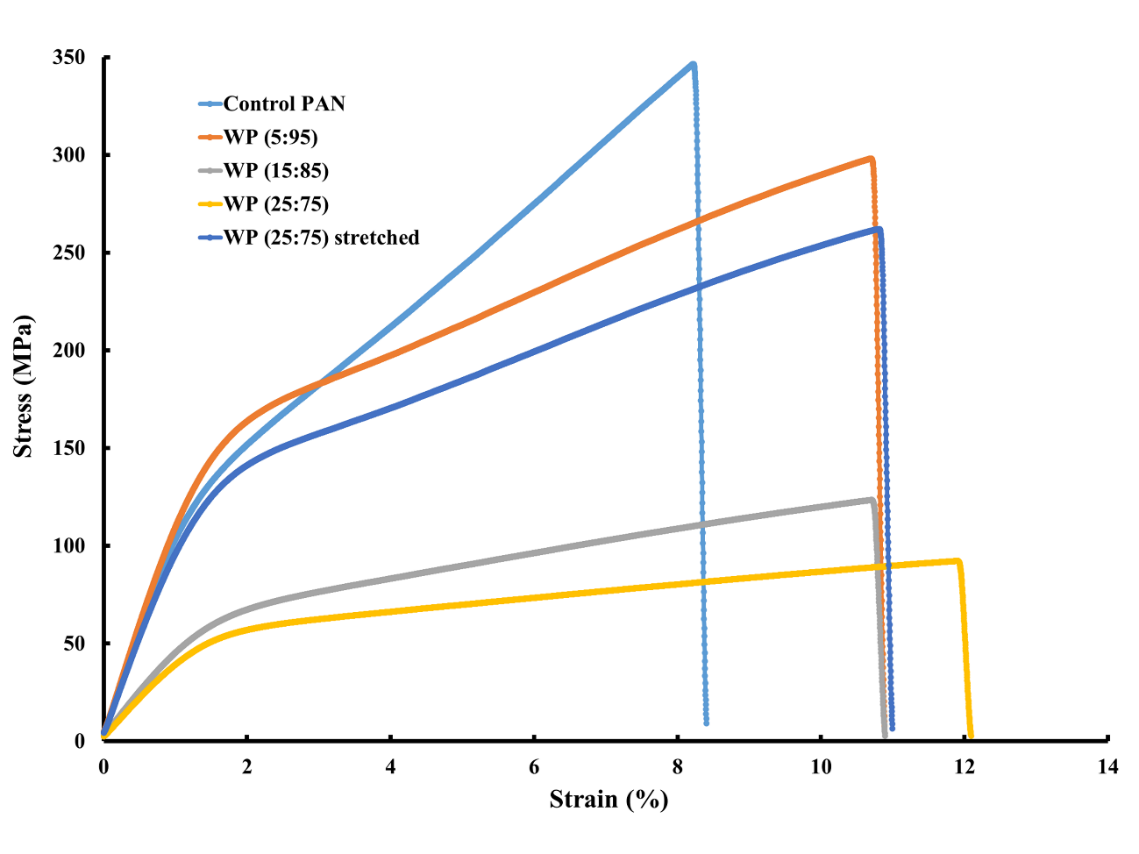


**Fig. S4.** Stress-strain curve of all the wet spun fibres.

**Table S1**

The value of K, n, and R^2^ after fitting the logarithmic curves of shear stress and shear rate.

| Sample | Consistency index (k) | Non-Newtonian index (n) | Correlation coefficient (R^2^) |
| --- | --- | --- | --- |
| Control PAN | 2.35 | 0.58 | 0.99 |
| WP (5:95) | 2.30 | 0.56 | 0.99 |
| WP (15:85) | 2.24 | 0.54 | 0.98 |
| WP (25:75) | 2.21 | 0.51 | 0.98 |

**Table S2**

The crystallinity index of control PAN and wool/PAN hybrid fibres.

| Sample | Peak intensity at 17.5⁰ (I_f_) | Peak intensity at 30.5⁰ (I_s_) | Crystallinity index (%) |
| --- | --- | --- | --- |
| Control PAN | 8294.56 | 2070.45 | 75.03 |
| WP (5:95) | 5465.19 | 1818.36 | 66.72 |
| WP (15:85) | 4869.26 | 2116.29 | 56.53 |
| WP (25:75) | 4260.61 | 2042.48 | 52.06 |

**Equation S1**

The Power-law equation was used to calculate the flow behaviour of the dope solutions ^6,7^.

σ = Kγ^n^  (S1)

where σ is the shear stress in Pascal, γ is the shear rate (1/s), K and n are the consistency index and non-Newtonian index, respectively ^6,7^. A linear curve fitting technique was employed using Origin 2019 software to obtain the regression parameters of the consistency index (K) and non-Newtonian index (n).

**Equation S 2-4**

The average crystallite size (L), as well as the interlayer spacing were calculated from the XRD spectrum for the GO powder using the Bragg’s Law ^8^ and Debye-Scherrer equation ^9^, respectively, as following:

Bragg’s law

nλ = 2dsin (ϴ) (S2)

where n is the diffraction order varies from 1, 2, 4 and λ is the X-ray wavelength, ϴ is the diffraction angle and the d is the spacing distance between two successive crystallographic planes (h, k, l) of the crystalline lattice (as described by Miller notation).

Scherrer's equation

L = k_s_λ/ (β cos (ϴ) (S3)

where L is the mean size of the ordered (crystalline) domains, k_s_ is a shape factor constant in the range 0.8–1.2 (typically equal to 0.9), λ is the X-ray wavelength, β is peak width at FWHM, θ is the Bragg angle.

The number of graphene layers (N) can be estimated through XRD analysis by correlating the crystallite size (L) with the interlayer spacing (d spacing) between the graphitic basal plans as discussed in the literature.

L = d (N-1) (S4)

**Equation S5**

The crystallinity index (Cr.I.) of the fibres was calculated by Equation S1:

Cr.I. = (I_f_ – I_s_) * 100 / I_f_ (S5)

where Cr.I. is the crystallinity index, I_f_ is the peak intensity in arbitrary units with 2-theta at 17.5⁰ and I_s_ is the peak intensity in arbitrary units with 2-theta at 30.5⁰ ^6,10^.

**Equation S6**

The resistivity of all the fibre samples was measured 20 times. The electrical conductivity of the CPrGO fibre and WPrGO hybrid fibre was calculated using the following formula ^11^.

$\sigma= \frac{L}{\pi r^{2}R}$ (S6)

where σ is the conductivity (S/cm), R is the resistivity (Ω), r is the radius (cm) and L is the length of the fibre (cm).

**References**

1 Al Faruque, M. A., Remadevi, R., Wang, X. & Naebe, M. Preparation and characterisation of mechanically milled particles from waste alpaca fibres. *Powder Technology* **342**, 848-855 (2019).

2 Jalili, R. *et al.* Scalable one‐step wet‐spinning of graphene fibers and yarns from liquid crystalline dispersions of graphene oxide: towards multifunctional textiles. *Advanced Functional Materials* **23**, 5345-5354 (2013).

3 Javed, K., Galib, C., Yang, F., Chen, C.-M. & Wang, C. A new approach to fabricate graphene electro-conductive networks on natural fibers by ultraviolet curing method. *Synthetic Metals* **193**, 41-47 (2014).

4 Liu, W.-w., Yan, X.-b., Lang, J.-w., Peng, C. & Xue, Q.-j. Flexible and conductive nanocomposite electrode based on graphene sheets and cotton cloth for supercapacitor. *Journal of Materials Chemistry* **22**, 17245-17253 (2012).

5 Pei, S. & Cheng, H.-M. The reduction of graphene oxide. *Carbon* **50**, 3210-3228 (2012).

6 Al Faruque, M. A., Remadevi, R., Razal, J., Wang, X. & Naebe, M. Investigation on structure and characteristics of alpaca-based wet-spun polyacrylonitrile composite fibers by utilizing natural textile waste. *Journal of Applied Polymer Science* **137**, 48370 (2020).

7 Al Faruque, M. A., Remadevi, R., Razal, J. M. & Naebe, M. Impact of the wet spinning parameters on the alpaca‐based polyacrylonitrile composite fibers: Morphology and enhanced mechanical properties study. *Journal of Applied Polymer Science* **137**, 49264 (2020).

8 Bragg, W. H. & Bragg, W. L. The reflection of X-rays by crystals. *Proceedings of the Royal Society of London. Series A, Containing Papers of a Mathematical and Physical Character* **88**, 428-438 (1913).

9 Holzwarth, U. & Gibson, N. The Scherrer equation versus the'Debye-Scherrer equation'. *Nature nanotechnology* **6**, 534-534 (2011).

10 El-Zaher, N. Study of the effect of ultraviolet radiation on some physical properties of dralon fabric. *Polymer-plastics technology and engineering* **40**, 689-702 (2001).

11 Xia, Y. & Lu, Y. Fabrication and properties of conductive conjugated polymers/silk fibroin composite fibers. *Composites Science and Technology* **68**, 1471-1479 (2008).
